# Attitude, knowledge, and predictors of COVID-19 vaccine uptake among health care providers in South Gondar public hospitals, North Central Ethiopia: multi-facility based study

**DOI:** 10.11604/pamj.2022.41.194.30868

**Published:** 2022-03-10

**Authors:** Solomon Demis Kebede, Tigabu Munye Aytenew

**Affiliations:** 1 Department of Pediatrics and Neonatal Nursing, Debre Tabor University, Tabor, Ethiopia,; 2 Department of Nursing, Debre Tabor University, Tabor, Ethiopia

**Keywords:** Health care providers, COVID-19 vaccine uptake, South Gondar, North Central Ethiopia

## Abstract

**Introduction:**

with this fast-increasing pandemic in terms of morbidity and mortality, all mankind is at risk of infection unless they get vaccinated and all African countries shall incorporate COVID-19 vaccination in their health care programs as long as the world work cooperatively. The pandemic, as the World Health Organization (WHO) stated, could be mitigated when 70% of the population which is nearly 5.6 billion should be immunized and to achieve this objective, the willingness of the community to be vaccinated before vaccination is essential.

**Methods:**

institutional-based cross-sectional study design was conducted from March 1^st^, 2021, up to 30^th^ May, 2021, in South Gondar Province, North Central Ethiopia. A structured interviewer-administered pre-tested questionnaire was used to collect data. The collected data were entered into EPI data version 4.2 and then exported into SPSS window version 22. Bivariate and multivariate analysis was undertaken and information was presented by using simple frequency tables and pie charts.

**Results:**

the majority of the respondents who accepted the COVID-19 vaccine accounted for nearly 260 (65%, 95% CI: 60-69). Positive attitude (AOR 2.5, 95% CI: 1.30-11.20), good knowledge (AOR 13, 95% CI: 6-27), bachelor of sciences (B.Sc) and above educational level (AOR 2.70, 95% CI: 1.30-6), TV or radio as source of information (AOR 0.10, 95% CI: 0.04-0.30), social media as source of information (AOR 0.04, 95% CI: 0.01-0.2), political leaders (AOR 0.08, 95% CI: 0.01- 0.90) were predictors of COVID-19 vaccine uptake.

**Conclusion:**

healthcare professionals (HCPs) with the decision (yes, sure) for COVID-19 vaccine uptake during data collection were found to be low as compared to other studies. Positive attitude, good knowledge, B.Sc, and above educational level were predictors enhancing COVID-19 vaccination uptake and TV or radio as a source of information, social media as a source of information and political leaders were factors decreasing COVID-19 vaccine uptake. Hence, it might be important to prioritize knowledge and attitude creation programs for HCPs and an alternative way of source of information and agents for the COVID-19 vaccine other than social media and religious leaders.

## Introduction

As the World Health Organization (WHO) defined novel coronavirus disease 2019 is an infectious disease caused by a newly discovered SARS-CoV-2 infection [1]. In African countries, the first case confirmed was on February 14^th^, 2020, announced in Egypt. And the first case confirmed in sub-Saharan countries was at the end of February. Within three months, the virus has spread throughout the continent of Africa. The impact of COVID-19 is more severe in the continent due to inadequate healthcare system in medical equipment, basic supplies like soap and water, health institutions in line with COVID-19, funds for COVID-19, and trained health professionals handling these emerging cases has shortage as of April 18^th^, 2020 [2]. The prevention and control system of the COVID-19 pandemic is not as effective as it was intended to be so in all parts of the world. It has been spreading globally and has affected all countries as global public health, socioeconomic and political threats by causing a significant challenge in morbidity and mortality all over the world. According to WHO 47 African countries has been affected with a total of 182,987,447 infected cases, and of 3,962,972 death globally and there were total cases of 5,554,549 and 143,360 deaths as of July 1^st^, 2021, in Africa [3]. The burden of the pandemic has influenced the nation directly and indirectly into all humankind. The direct impact includes mental health disturbances by fearing illness and death secondary to the COVID-19 and societal lifestyle changes the policymakers implemented indirectly [4]. Even though COVID-19 vaccination has been administered nowadays in an increasing manner by doses day by day, there were vaccine hesitancy reports in African countries including Ethiopia where the rate of morbidity and mortality is among the 5^th^ leading countries in Africa [5]. Thus, it indicates that prevention of COVID-19 transmission in developing countries in general and in Ethiopia specifically is not effective as it has intended earlier. No other choice nowadays left to curve the effect of the pandemic than delivering vaccination in the community primarily to health care providers [5].

Africa has ever-increasing derive to vaccination is now underway with inline WHO recommendation in the fight against the pandemic as a future strategy. Hence, there have been research to produce a vaccine to control the pandemic worldwide, by which vaccine for COVID-19 is available in almost all African countries. With this fast-increasing pandemic in terms of morbidity and mortality, all mankind is at risk of infection unless they get vaccinated and all African countries shall incorporate COVID-19 vaccination in their health care programs as long as the world works cooperatively. The pandemic, as the WHO stated, could be mitigated when 70% of the population which is nearly 5.6 billion should be immunized and to achieve this objective, the willingness of the community to be vaccinated before vaccination is essential [6,7]. However, there were reported challenges towards COVID-19 vaccination as traditional medicine and sociocultural factors might play a role by which it is widely acceptable and accessible in African countries compared to modern therapies including vaccination packages in health care settings. Traditional medicine has been serving the modern health care system as a complement and part of the care, which is accepted and recognized as a cultural practice. Even with the emerging modern medicine, the traditional indigenous health care delivery system is the major source of care estimated at 80% of the population in Ethiopia [8,9]. The world is relying on the vaccines to control effectively and efficiently from AstraZeneca/Oxford, Johnson and Johnson, Moderna, Pfizer/Biotech, Sinopharm, and Sinovac granted by WHO for emergency use. These vaccine producers recommend that health care providers should get COVID-19 vaccine as they are at higher risk of exposure to the virus [7,10]. The health care providers have been engaged in multiple responsibilities with the era of amid COVID-19 pandemic. They are expected to deliver health education about COVID-19 vaccination, which can be effective if they are willing to be vaccinated. Hence, identifying their vaccine uptake status would be important. No previous studies explored predictors of COVID-19 uptake among health care providers in Ethiopia by whom they would provide health education to the community about COVID-19 vaccine, health promotion, and disease prevention programs.

## Methods

**Study design:** a health institution-based cross-sectional study design was conducted from April 1^st^ to May 30^th^, 2021 in South Gondar public hospitals. There are eight public hospitals in the zone namely Debre Tabor Comprehensive and Specialized, Addis Zemen, Ebnat, Mekane-Eyesus, Andabet, Wogeda, Nefas Mewucha, and Tach Gayint hospitals. It has also 93 health centers and 378 health posts. The health coverage in the zone is 89.95% at the health post and 100% at the health center level. The health care providers in the South Gondar Province working in public hospitals during the data collection period were included. Simple random sampling techniques were used with the assumption of similar sociocultural characteristics of the health professionals, their educational background, and background knowledge to the COVID-19 pandemic.

**Sample size:** the sample for each hospital was taken using proportional to probability to size (PPS) samples as follows. Debre Tabor Comprehensive and Specialized Hospital (n=178), Addis Zemen Hospital (n=45), Ebnat Hospital (n=31), Mekane-Eyesus Hospital (n=38), Andabet Hospital (n=35), Wogeda Hospital (n=30), Nefas Mewucha Hospital (n=36) and Tach Gayint Hospital (n=29). Using the single proportion formula:


$$N=\frac{{\left(Z\alpha /2\right)}^{2}P(1-P)}{d^{2}}$$

Where: N= the minimum sample size required for the study; Z= standard normal distribution (Z=1.96) with 95% confidence interval; P= COVID-19 vaccine uptake proportion (P= 50%); d= is a tolerable margin of error (d= 5%), the sample size (N) was 422.

**Data collection tool and procedure:** the structured self-administered questionnaire was used to collect the data developed from previous pieces of literature [6,11-16]. The questionnaires include: 1) sociodemographic characteristics of the respondents; 2) source of information about COVID-19; 3) trusted agents for COVID-19 vaccine; 4) perceived severity of the COVID-19; 5) attitudes towards vaccination with Likert scale of a total of 14 questions; 6) knowledge with multiple-choice questions about standard current COVID-19 vaccine information. The data collectors checked for missing data on spot at the session of data collection time. The title of the study, the objective of the study, the time needed to respond to the questions, the rights of the respondents, and their consent form are attached to the questionnaire. The respondents before the start of the data collection were informed and asked for signed consent.

**Dependent variable:** the dependent variable of the study was the uptake of the COVID-19 vaccine. Those respondents answered the question “are you going to take COVID-19 vaccine now at this responding time?”. If the answer was 'yes sure´ and 'yes may be´ classified as uptake group and those responded 'no´´ and 'I don´t know´ to the did not uptake group [6].

**Method of data analysis:** the mean and standard deviation for age category; frequency tables and pie charts for categorical variables. Chi-square test to identify the relationship of categorical variables. Binary logistic regression model to identify the predictors of COVID-19 vaccination uptake (vaccine uptake group versus did not uptake group) with odds ratio (OR) probability and 95% CI. The statistical analysis was performed using IBM SPSS version 22 with statistical significance was declared at p-value <0.05.

**Ethical approval:** it was obtained from Debre Tabor University, college of health sciences, Institutional Health Research Ethics Review Committee (IHRERC 2021). Informed consent was obtained from each study participants.

## Results

**Sociodemographic characteristics of the respondents:** the response rate of this study was (402) 95%. The mean age and standard deviation (SD) of the respondents was 29±4.45. The majority were married, B.Sc and above, and nurse professionals accounted for 203 (50.5%), 298 (74%) and 284 (70.6%) respectively (Table 1).

**Table 1:** sociodemographic characteristics of the respondents

Variable	Category	Frequency (%)
Sex	Male	225 (56)
	Female	177 (44)
Marital status	Single	193 (48)
	Married	203 (50.5)
	Others	6 (1.5)
Age	≤30 years	260(64.7)
	>30 years	142(35.3)
Educational qualification	Diploma	104(25.9)
	BSc and above	298(74.1)
Profession	Nurse	284(70.6)
	Medical doctor	74(18.4)
	Others	44(11.0)

**Source of information about COVID-19 vaccine:** the majority of them got information from TV or radio accounted for nearly 184 (46%) and the least of them from social media accounted for nearly 34 (9%). The most trusted agents in providing COVID-19 vaccine information were spiritual leaders (Qes or shekh) accounted for nearly 238 (59%) (Table 2).

**Table 2:** source of information about COVID-19 vaccine

Variables	Category	Frequency (%)
Source of COVID-19 vaccine	Written materials	66 (16.4%)
	TV or radio	184 (45.8)
	Social media	34 (8.5)
	Official pages of health organizations	58 (14.4)
	Google search	60 (14.9)
The most trusted agents for COVID-19 vaccine	Spiritual leaders (Qes or Shekh)	238 (58.7)
	Journalists or bloggers	82 (20.4)
	Politicians	33 (8.2)
	Not all trusted	31 (7.7)
	Others	20 (5.0)

**Figure 1:**
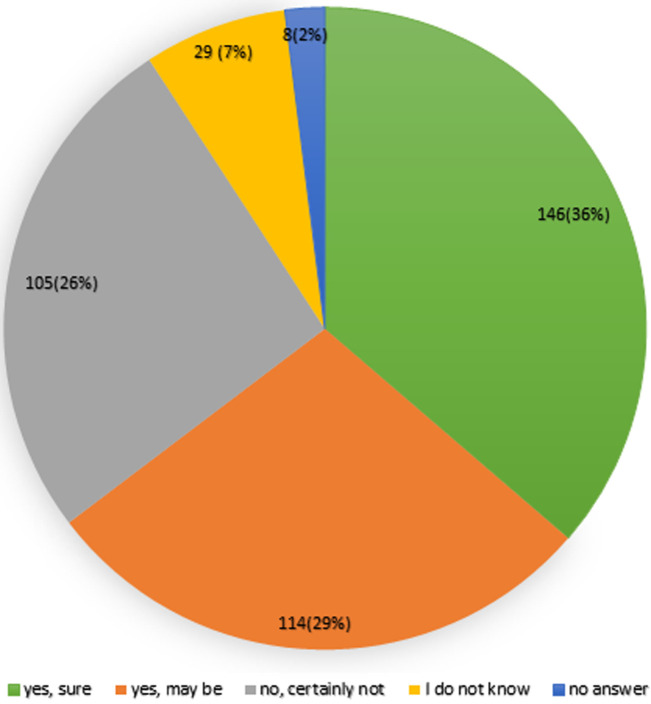
all the responses of COVID-19 vaccine uptake status during the data collection period at South Gondar public hospitals, North Central Ethiopia, 2021

**COVID-19 vaccine uptake:** the majority of the respondents' uptake of COVID-19 vaccine accounted for nearly 260 (65%, 95% CI: 60-69) and the lesser was 142 (35%). Regarding, COVID-19 vaccine uptake status among respondents during the data collection period showed that the majority of them were surely voluntary and may be voluntary to be vaccinated which accounted for 146 (36.32%) and 114 (28.36%) respectively by which respondents were voluntary to take the vaccine during data collection and the least were those who did not want to respond accounting for 8 (1.99%) (Figure 1).

**Figure 2:**
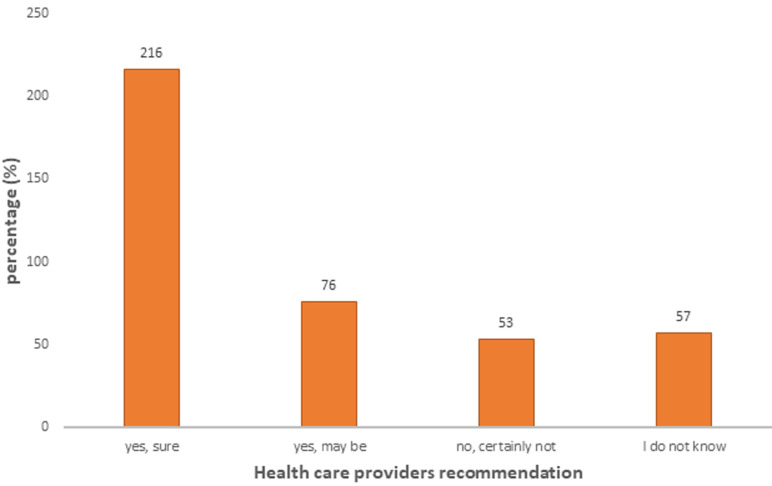
health care providers´ recommendation to COVID-19 vaccine to their clients at South Gondar public hospitals, North Central Ethiopia, 2021

**Recommendation to their clients:** when the COVID-19 vaccine was available at the zone, how health care providers recommend and encourage their clients to be vaccinated indicated that the majority of the respondents were those who would have encouraged their clients to be vaccinated accounted for 216 (53.73%) and the least of them were those who would have not to recommend for vaccination which accounted for 53 (13.18%) (Figure 2).

**Figure 3:**
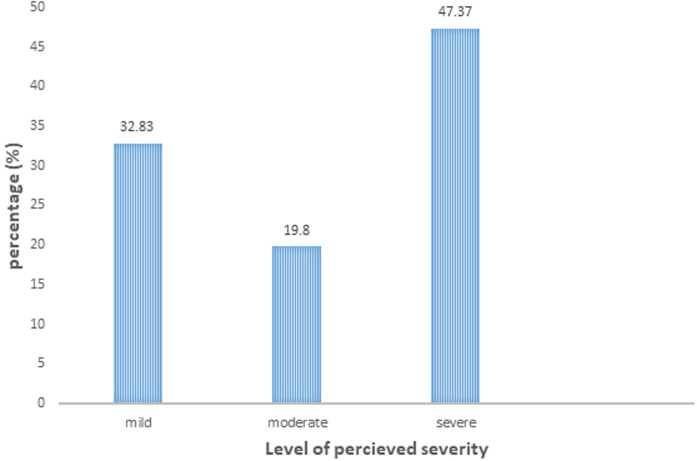
perceived severity of COVID-19 among HCPs at South Gondar public hospitals, North Central Ethiopia, 2021

**Perceived severity for COVID-19 infection:** the majority of the respondents, regarding the severe perceived severity for COVID-19 infection, were 192 (47.76%) and the least were those with moderate perceived severity accounted for 79 (19.65%) (Figure 3).

**Knowledge towards COVID-19 vaccine:** more than two fold of the respondents who had good knowledge accounted for 273 (68%, 95% CI: 52-74), and those with poor knowledge were 129 (32%, 95% CI: 23-42).

**Predictors of COVID-19 vaccine:** the perceived severity of COVID-19, sex of the respondents, age category, level of education, profession, source of information from political leaders, attitude towards COVID-19 vaccine, and knowledge of the COVID-19 vaccine was associated with COVID-19 vaccine uptake in univariate analysis. The variables whose P-value were found as <0.10 in univariate analysis were included in multivariate logistic regression analysis with entering method. P-value <0.05 was accepted as statistically significant. Positive attitude (AOR 2.5, 95% CI: 1.30-11.20), good knowledge (AOR 13, 95% CI: 6-27), B.Sc and above educational level (AOR 2.70, 95% CI: 1.30-6), TV or radio as a source of information (AOR 0.10, 95% CI: 0.04-0.30), social media as a source of information (AOR 0.04, 95% CI: 0.01-0.2), political leaders (AOR 0.08, 95% CI: 0.01- 0.90) were associated with COVID-19 vaccine uptake in multivariate logistic regression (Table 3).

**Table 3:** predictors for COVID-19 vaccine uptake among health care providers in South Gondar Public Hospitals, 2021

	COVID 19 Vaccine uptake status		95% CI	
	Uptake	No uptake	COR	AOR
**Attitude**				
Positive attitude	238(72.6%)	90(27.4%)	6.25(3.60, 10.80)	2.5 (1.30, 11.20)***
Negative attitude	22(29.7%)	52(70.3%)	1	1
**Knowledge**				
Good	115(55%)	94(45%)	2.46(1.60, 3.80)	13 (6, 27) ***
Poor	145(75%)	14648(25%)	1	1
**Educational level**				
BSc and above	185(62%)	113(38%)	0.63(0.010, 0.37)	2.70(1.30, 6) **
Diploma	75(72%)	29(28%)	1	1
**Source of information**				
TV or radio	105(57)	79(43)	2.47(1.27,4.80)	0.10(0.04, 0.30)
Social media	3(9)	31(91)	33.95(9,128)	0.04(0.01,0.2)
Google search	46(77)	14(23)	1	1
**The trusted source of information**				
Political leaders	3(9.9)	30(91)	13.84(3.50,55)	0.55(0.12,2.70)
Religious leaders	11(95)	9(5)	0.07(0.009,0.65)	0.08(0.01, 0.90)**
Not all trusted	18(58)	13(42)	1	1

COR: crude odds ratio; AOR: adjusted odds ratio, CI: confidence interval; ** p-value <o.o5, *** p- value < 0.001

## Discussion

**COVID-19 vaccine uptake status:** COVID-19 vaccine uptake status was found to be nearly 260 (65%, 95% CI: 60-69). This finding was comparable with studies in Turkey, Italy, and China ranging from 49.7 to 67% [15-17]. And this finding was higher compared to studies in Turkey, the UK, Taiwan, and India ranging from 14% to 45.5% [4,18]. This might be due to variation in data collection method and study period. In this study, those who were surely voluntary to be vaccinated and may be voluntary were considered as those who would be vaccinated. This might increase the uptake group compared to other studies. This finding was lower than studies conducted in China, France, and Israel, ranging from 78% to 91.3% [19-21]. This might be due to variation in sample size, data collection method, and study period. Regarding the HCP recommendation for COVID-19 vaccine to their clients was nearly 216 (54%, 95% CI: 48.5-58.7). This indicated that nearly half of the HCPs were reluctant to encourage to give health information about the COVID-19 vaccine to their clients. This finding was lower than studies conducted in France and French-speaking people [21]. This difference might be explained by sample size, study period at the highest surge of pandemic, and method of data collection. Positive attitudes towards the COVID-19 vaccine was found to be 54% which was higher than the study in Tigray about COVID-19 pandemic. Good knowledge about COVID-19 vaccine in this study was 273(68%, 95% CI 52-74) which was lower than studies in Tigray about the COVID-19 pandemic [22].

**Predictors of COVID-19 vaccine uptake:** those HCPs with a positive attitude towards the COVID-19 vaccine were 2.5 times more likely to uptake the COVID-19 vaccine than those with a negative attitude. This could be explained that HCPs with positively inclined attitudes towards COVID-19 vaccine would have been devoted to the new update, and they might clarify and confusion through trusted media like WHO, Federal Ministry of Health (FMoH), electronic protected health information (EPHI), and the like. On the contrary, the HCPs with a negative attitude towards COVID-19 vaccine were exposed to the previous unclear set of information without updating and searching for new evidence timely. They kept their previous doubt irrespective of the current time and evidence explored. Those with good knowledge about COVID-19 vaccine were 13 times more likely to uptake the COVID-19 vaccine than those with poor knowledge. This might be explained as being knowledgeable about the COVID-19 vaccine allows differentiating the truth evidence worldwide from the myths. Healthcare professionals with good knowledge could evaluate the evidence through scientific pieces of evidence currently available. This in turn helps them to have their stand towards the newly produced COVID-19 vaccine. Those with BSc and above educational level were 2.7 times more likely to uptake COVID-19 vaccine than those with diploma educational level. This might be since HCPs with BSc and above educational level had higher access to new information through their academic merits, such as being closer to the scientific community while doing their graduation thesis in university. In other words, those diploma holders were, more or less, in district parts of the health facility where there was limited access to health information through the internet by google search. Those who had COVID-19 vaccine information from TV/radio and social media were 1% and 4% less likely to uptake COVID-19 vaccine than those who got information from Google search. This might be since those who googled about the vaccine were eager to get vaccine updates purposefully. They might be intentionally accessed the internet to clear their doubt about the COVID-19 vaccine. And respondents who trusted religious leaders at most were 8% less likely to uptake the COVID-19 vaccine than those who did not trust all agents. This might be the fact that religious leaders in Ethiopia were inclined to their spiritual healing process and HCPs trusted that way of newly emerging pandemic stayed late to decide on scientific solutions like COVID-19 vaccine uptake.

## Conclusion

The COVID-19 vaccine uptake status (yes sure and yes maybe) among health care providers was found to be low as compared to other studies. Healthcare professionals with the decision (yes, sure) for COVID-19 vaccine uptake during data collection were the least among other studies. Positive attitude, good knowledge, BSc, and above educational level were predictors enhancing COVID-19 vaccination uptake and TV or radio as a source of information, social media as a source of information and political leaders were factors decreasing COVID-19 vaccine uptake. Hence, it might be important to prioritize knowledge and attitude creation programs for HCPs and an alternative way of source of information and agents for the COVID-19 vaccine other than social media and religious leaders.

### What is known about this topic

- There have been COVID-19 vaccine hesitancy among health care workers in various countries in the world;
- Research findings in various countries about COVID-19 vaccine hesitancy were inconsistent.

### What this study adds

- This study is the first to measure COVID-19 vaccine uptake in South Gondar zone public hospitals specifically and in Ethiopia at large; hence, it can be used as baseline evidence for further study;
- It has identified predictors either enhancing or hindering health professionals' COVID-19 vaccine uptake;
- It has described the status of knowledge and attitudes of health professionals towards the COVID-19 vaccine in South Gondar zone public hospitals North Central, Ethiopia.
